# P01 Catastrophic antiphospholipid syndrome presenting as diffuse peripheral and central thromboses

**DOI:** 10.1093/rap/rkab068

**Published:** 2021-10-19

**Authors:** Saad Ahmed, Dana Low, Tanjina Nasrin, Lauren Steel

**Affiliations:** Colchester Hospital, Colchester, United Kingdom

## Abstract

**Case report - Introduction:**

Catastrophic Antiphospholipid Syndrome (cAPS) is the most severe form of antiphospholipid syndrome with a high mortality; it is characterised by multiorgan involvement that develops within a short time frame and usually consists of microvascular thrombosis. We present the case of a 50-year-old lady with recurrent microvascular and macrovascular thromboses who was initially treated with endovascular stents and amputation progressing to require immunosuppression and anticoagulation, to include steroids, rituximab, intravenous immunoglobulin and plasma exchange.

**Case report - Case description:**

A 50-year-old lady presented to the Emergency Department with bilateral leg and abdominal pain. Her co-morbidities included type two diabetes, psoriasis, three miscarriages, borderline personality disorder and a heavy smoking history. Clinical examination revealed pulse deficits in the distal lower limbs with gangrene evidence of ischaemia on her toe digits. CT angiogram demonstrated complete thrombus of the Infrarenal abdominal aorta extending to the common iliac and external iliac arteries bilaterally. Thrombolysis ensued and an aortic stent was inserted with symptom relief. Two weeks later readmission occurred with bilateral leg pain; Ultrasound Doppler revealed a tight stenosis at the distal aortic region. Initial management consisted of Intravenous heparin but worsening ischaemia resulted in insertion of kissing stents at the aortic bifurcation. The patient’s pain settled with no residual arterial compromise. One month later the patient was re-admitted with bilateral leg pain and necrotic right toes; this led to a right forefoot amputation. A triphasic bilateral finger colour change was noted with ischaemic pain and livedo reticularis on lower limbs, with a decision to institute Iloprost and methylprednisolone ensued. Antiphospholipid antibodies returned showing triple positivity. Management subsequently included addition of IV rituximab, plasma exchange, IVIG and sildenafil. Two months later the patient was re-admitted with complete lower limb paralysis due to a complete thrombus of the aortic bi-iliac stent; thrombolysis ensued with good result. A further admission 1- month later occurred due to sepsis and an infected necrotic left forefoot resulting in an above knee amputation. No further endovascular stenting was advised to risk of embolic seeding following medical management.

**Case report - Discussion:**

We have described a case of cAPS on a previously asymptomatic female patient who presented with diffuse peripheral and central thromboses. Our patient suffered from intra-abdominal organ infarction and subsequent acute kidney injury, recurrent arterial and venous occlusion over a period of 12 months and previous pulmonary emboli. Livedo reticularis and gangrene were visible cutaneous manifestations of this disorder on our patient. cAPS accounts for less than 1% of APS and has a high mortality of 50% which means early and frequent discussion with specialist centres is important.

In addition to the clinical features described in our patient, laboratory features included moderate thrombocytopaenia and evidence of haemolysis (raised bilirubin and LDH). The cAPS registry demonstrates that the majority of patients are female (72%) with a mean age of 37 years, 46% have primary APS, 40% suffer from SLE and 9% from other autoimmune diseases. This patient does not have a secondary autoimmune condition.

The most common clinical features to present before cAPS develops include foetal loss, previous DVT or thrombocytopaenia, two of which our patient demonstrated. The prognosis and clinical features of cAPS have been shown to depend on the extent of thrombosis, organs affected and the presence of a systemic immune response from affected tissues. Treatment options available for cAPS consist of multiorgan support, anticoagulation and immunosuppression, in the form of glucocorticoids, rituximab, IV Immunoglobulin and plasma exchange. Our patient required all of these due to accelerating thrombosis as determined by new gangrene, ongoing livedo reticularis rash and thrombocytopenia.

**Case report - Key learning points:**

Our case demonstrates the importance of keeping a high index of suspicion for cAPS as up to 46% will have this as their first presenting feature of APS — including our patient. On her admission to hospital particular attention was paid to clinical examination which suggested Raynauds and a skin rash consistent with livedo reticularis — this prompted a rheumatology consult, serology testing, starting Iloprost and tertiary centre transfer. The diagnosis was secure with high titre of IgG anticardiolipin antibody, anti Beta-2 glycoprotein 1 antibodies and Lupus Anticoagulant detected — all on two occasions more than 12 weeks apart.

Patients may present to surgical specialties in view of peripheral vascular symptoms and signs. It would be appropriate to identify patients with APS early to prevent multiple surgeries or considerations for endovascular stents, as they are frequently not successful. This case highlights the need for discussion and education within the multi-disciplinary setting for patients with APS, including surgical teams.

Finally, the risk of immunosuppression for patients who have received rituximab can persist for up to 12 months following treatment and this lady also had the co-morbidity of diabetes. This patient’s risk stratification was high in view of COVID-19 and she was advised to shield until government guidelines ended last year. Currently she is doing well without new symptoms and she will be reviewed at 6 months following rituximab, in the Autumn of 2021.

